# Nurse competence in the post-anaesthesia care unit in Sweden: a qualitative study of the nurse’s perspective

**DOI:** 10.1186/s12912-021-00792-z

**Published:** 2022-01-05

**Authors:** Karuna Dahlberg, Ann-Sofie Sundqvist, Ulrica Nilsson, Maria Jaensson

**Affiliations:** 1 School of Health Sciences, Faculty of Medicine and Health, Örebro University, 701 82 Örebro, Sweden; 2 University Health Care Research Center, Faculty of Medicine and Health, Örebro University, Örebro, Sweden; 3 Department of Cardiothoracic and Vascular Surgery, Örebro University Hospital, Örebro, Sweden; 4 Division of Nursing, Department of Neurobiology, Care Sciences, and Society, Karolinska Institutet, and Perioperative Medicine and Intensive care, Karolinska University Hospital, Stockholm, Sweden

**Keywords:** Competence, Education, Nursing, Post-anaesthesia care unit, Postoperative period

## Abstract

**Background:**

To enable safe and successful recovery for surgery patients, nurses working in post-anaesthesia care units need competence in postoperative care. No consensus defines what this specific competence includes, and it has not been studied from the perspective of nurses working in post-anaesthesia care units. The aim of this study is twofold: 1) To explore and describe nurses’ perception of the competence needed to work in post-anaesthesia care units. 2) To explore and describe nurses’ perception of what characterizes an expert nurse in post-anaesthesia care units.

**Methods:**

This qualitative inductive study uses individual interviews. Sixteen nurses were recruited from two post-anaesthesia care units located in different parts of Sweden. Inclusion criteria were nurses employed in the post-anaesthesia care units for ≥1 years.

Semi-structured individual interviews were conducted; data were analysed using thematic analysis.

**Results:**

The interview analysis identified six subthemes and three themes. The themes *being adaptable in an ever-changing environment* and *creating safe care* represent the overarching meaning of competence required when working as a nurse in a Swedish post-anaesthesia care unit. Nurses must possess various technical and nontechnical skills, which are core competences that are described in the sub-themes. The theme *seeing the bigger picture* describes the nurse’s perception of an expert nurse in the post-anaesthesia care unit.

**Conclusions:**

Nurse competence in post-anaesthesia care units entails specific knowledge, acknowledging the patient, and working proactively at a fast pace with the patient and team to provide safe, high-quality care. An expert nurse in post-anaesthesia care units can see the bigger picture, helping share knowledge and develop post-anaesthesia care. The expert competence to see a bigger picture can be used in supervising novices and creating a knowledge base for postgraduate education in order to promote safe, high-quality post-anaesthesia care.

## Background

There is inconsistency in definitions of nurse competence, competence can be defined as the “possession of required skill, knowledge, qualification, or capacity” [1] or “a skill that you need in a particular job or for a particular task” [2]. In a concept analysis from 2012, nurse competence was described as integrating knowledge into practice, experience, critical thinking, proficient skills, caring, communication, environment, motivation, and professionalism [3]. The International Council of Nurses defines nurse competence as comprising knowledge, skills, and judgement [4]. It is important to note that nurse competence is not a goal but a journey, consistent with Benner’s *novice to expert* model, which describes five levels of competence based on knowledge and skill acquisition [5]. As nurses acquire deeper knowledge and skills, they move through five competence levels: novice, advanced beginner, competent, proficient, and expert. Competence is situational and context specific, and highlighting and defining the expert nurse level clarifies for nurses at lower competence levels what to strive for to attain the expert nurse level of competence [5].

Nurse competence differs between contexts, and in the pre-, intra-, and postoperative contexts it involves both technical and nontechnical skills [6–9]. To enable safe and successful recovery for patients who have undergone surgery, nurses need competence in postoperative care [10, 11]. However, there is no consensus regarding the competence or education needed to provide safe, high-quality patient care in post-anaesthesia care units (PACU) [12, 13]. This lack of consensus has been raised and discussed by the ICPAN, International Collaboration of PeriAnesthesia Nurses board and its members and is an area that needs further development and collaboration to strengthen nursing in the PACU [14, 15].

In Sweden most nurses working in PACUs are specialist nurses in intensive care, i.e., registered nurses (RNs) with an additional postgraduate diploma (60 credits in second-cycle higher education, masters level) [13, 16]. However, there is no specific postgraduate education in postoperative care. It is therefore important to explore and describe how nurses describe competence in postoperative care and what they experience as necessary to be able to work in PACUs. This knowledge is important in order to enhance safe care and to develop postgraduate education in postoperative care. To this end, the aim of this study is twofold: 1) To explore and describe nurses’ perception of the competence needed to work in PACUs. 2) To explore and describe nurses’ perception of what characterizes an expert nurse in PACUs.

## Methods

This qualitative inductive study with individual interviews was conducted from September 2019 to January 2020.

### Setting and participants

Nurses were recruited from two PACUs located in different parts of Sweden. Both PACUs were open 24 h a day 7 days a week, providing both preoperative and postoperative care to inpatients and outpatients. Detailed information about the PACUs is presented in Table 1.

**Table 1:** Characteristics of the included PACUs

	PACU 1	PACU 2
Type of hospital	District hospital	University hospital
Number of beds (during office hours)	40	42
Number of nurses employed	31	23
Other professionals stationed in the PACU besides nurses	Nurse assistant^a^	Nurse assistant^a^ Anaesthesiologist

*PACU* Post-anaesthesia care unit

^a^Has an upper-secondary qualification and works under the supervision of a nurse

Nurses were informed orally about the study by the first author at a workplace meeting. Additional written information was sent to all the nurses (*n* = 54) by e-mail, so all of them would receive information about the study even if they had not attended the meeting. Convenience sampling was conducted, i.e., nurses interested in participating contacted their PACU manager or the first author. Inclusion criteria were nurses employed in the PACU, with ≥1-year experience from working in the PACU. The aim was to recruit nurses with variation in duration of PACU work, education level, and sex.

### Data collection

A total of 16 nurses (eight from each PACU) were interested in participating and all of them were interviewed and included in the analysis (Table 2).

**Table 2:** Overview of included participants

Female/male, *n*	14/2
**Years of experience as a graduated nurse**	
Mean	21.8
Median	18.5
Min–max	7–46
**Years of PACU work**	
Mean	7.8
Median	4.5
Min, max	1, 35
**Type of education,*n***	
Nurse without postgraduate diploma	3
^a^*Specialist nurse in:*	
• Intensive care	7
• Acute care	4
• Anaesthetic care	3
• Paediatric care	1
• Surgical care	1

*PACU* Post-anaesthesia care unit

^a^Nurses with an additional post-graduate diploma in nursing, 60 credits; three nurses had two postgraduate diplomas

Individual interviews were conducted by the first author and they ended when no new information was forthcoming. A semi-structured interview guide (Table 3) was used during the interviews and the areas of interest were probed to obtain deeper and richer data. The first interview served as a pilot interview to test the interview guide. Since no revisions of the guide were required, this interview was included in the analysis. The interviews were conducted using an inductive approach, which is appropriate given the limited research in this context. The participants decided where they wanted the interviews to be conducted. Fifteen interviews were conducted face to face at the participants’ workplaces and one interview was conducted over the telephone. Participants were released from work or came in their spare time and interviews were conducted in a room secluded from the ward. There were no disturbances during the interviews. The interviews lasted 36–90 min (mean, 55 min). All interviews were recorded and transcribed verbatim by a professional transcriptionist, yielding a total of 242 single-spaced pages that formed the basis of the analysis. The accuracy of the transcripts was verified by the first author through a process of concurrently reading and listening to the recorded interviews.

**Table 3:** Interview guide

Describe the nurse’s role in PACU care.	
What is unique about a nurse working in the PACU versus in a ward or the operating room?	
What competence is needed by nurses working in the PACU?	
- specific skills?	
- specific education?	
What characterizes an *expert nurse* working in the PACU?	
- What does he or she know/do that others don’t know/do?	
What characterizes safe and secure care in PACU?	
What situations in the PACU are the most challenging?	
- give examples	

### Data analysis

The interviews were analysed using thematic analysis described by Braun and Clarke [17]. The analysis started with the first and second authors reading and rereading the transcribed interviews to become familiar with the data. The other two authors read seven interviews each to get a sense of the core of the interviews. After the interviews were read several times, the interviews were coded and the codes were then searched for patterns and themes in relation to the two aims. The coding and initial sorting of sub-themes and themes was done independently by the first and second authors and then jointly discussed and refined. The emergent themes and sub-themes were then reviewed by the other two authors, who had not been part of the initial analysis, to confirm the findings. All four authors then discussed the themes and sub-themes to ensure correspondence with the original data and study aim as well as the interpretation of the data. Throughout the analysis, the first two authors moved between the different phases of the analysis in an iterative process. Examples of the data analysis are presented in Table 4.

**Table 4:** Examples of the data analysis

Data extract	Code	Sub-theme	Theme
*because even if you have great colleagues and so on, you still have to make pretty quick decisions and...know how to navigate the system, know how you’re supposed to think and so on.*	Independent – quick decisions	To be independent	**Being adaptable in an ever-changing environment**
*But often you do know what’s going on and people come and ask, that’s the thing, so I feel that – I suppose that’s also why, I always try to answer questions, give them what they need, because...I think it’s better they come and ask so we all help each other out. But sometimes it’s, of course it’s challenging, I mean sometimes it’s hard to keep up with everything.*	Tries to help others and answer questions even when has a lot to do	To work jointly in a team	
*they get a minor shock when the evening comes and...patients start to pour in and they all have VAS 8 and are feeling awful, then it’s no longer just that simple anymore; you have to...be able to prioritize and work pretty quickly if you want everything to work out well.*	Much to do, prioritize	To prioritize and make clinical decisions in a fast pace	
*Safe. Yeah, but it’s like we talked about, all this, I think, I mean let the patient be involved, the patient has to be a part of his or her care. Not exclude the patient, I mean, you have to involve the patient all the time. Yes...*	The patient is part of the care, creates a sense of safety	To acknowledge and reassure the patient	**Creating safe care**
*And here it’s a whole other type of pain relief in a post-anaesthesia care unit, so I found it a bit challenging at first, I must say. I thought “Oh dear, how am I supposed to know how much morphine and what should I supplement with it?*	Specialist knowledge in pain	To possess specific knowledge	
*And you’re prepared for a possible unpleasant surprise? Absolutely, yes, yes. And it’s the same when, say, you have to re-do the epidural and so on, so if there’s no noradrenaline I make sure there’s a syringe containing noradrenaline that’s connected, basically I make sure I’m prepared.*	Prepared for possible complications	To anticipate the future and be proactive	
*You’re like someone who has many answers to questions to those who are...students and new colleagues at a workplace...succeed in teaching them this stuff, basic PACU nursing and not specific to different operations but basic PACU nursing*	Expert, sees new colleagues can teach others		**Seeing the bigger picture**

### Ethical perspective

The study followed the principles outlined in the Declaration of Helsinki and was approved by the Swedish Ethical Review Authority (reference number: 2019–02615). Participants received written and oral information about the study and were informed that participation in the study was entirely voluntarily. The participants gave their written informed consent before the interviews took place. The researcher conducting the interviews had no previous or ongoing experience of the included PACUs, i.e., had no pre-understanding of the units, to ensure that the participants would speak freely about the topic. All recorded interviews and transcripts are stored in a secure place only available to the researchers.

## Findings

**Fig. 1:**
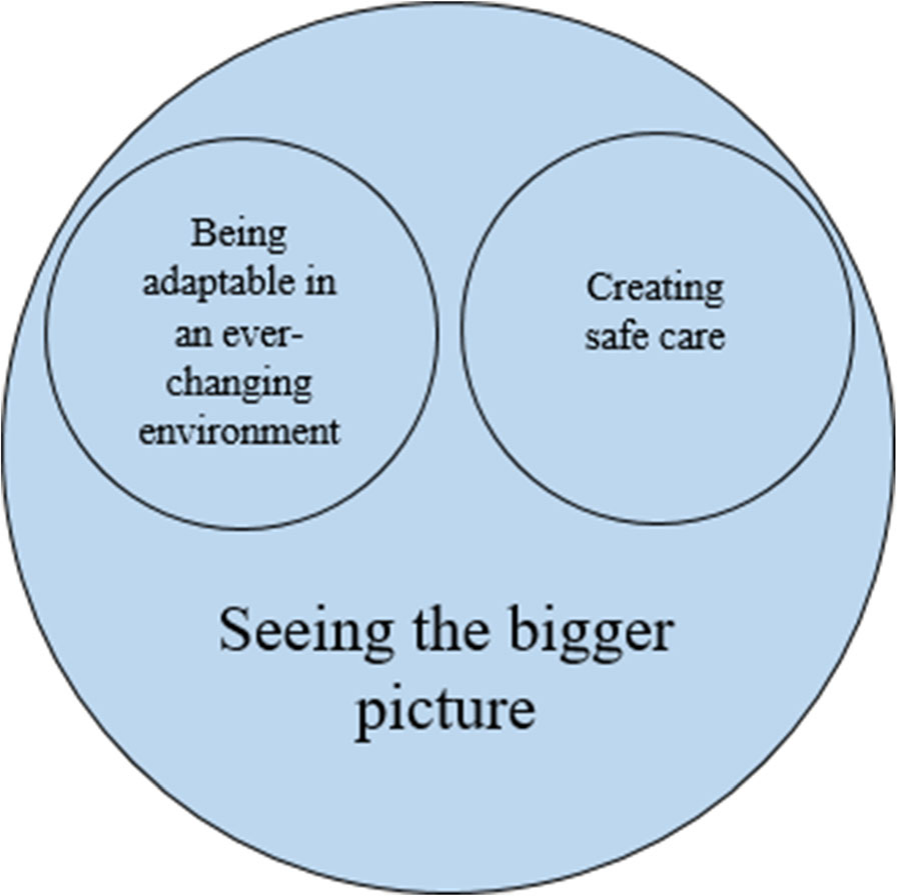
An overview of the theme *Seeing the bigger picture* that describes being an expert PACU nurse. To see the bigger picture nurses have to possess all of the required knowledge and unique skills as described in the themes *being adaptable in an ever-changing environment* and *creating safe care*

The analysis of the interviews identified three themes and six subthemes. The themes *being adaptable in an ever-changing environment* and *creating safe care* represent the overarching meaning of competence that is required when working as a nurse in Swedish PACUs. Nurses must possess various technical and nontechnical skills, which are core competences that are described in the sub-themes. The theme *seeing the bigger picture* describes the nurse’s perception of an expert nurse in the PACU. An overview of the themes and sub-themes is shown in Table 4 and Fig. 1.

### Being adaptable in an ever-changing environment

Working as nurse in a PACU entailed being adaptable in an ever-changing environment, which was characterized by the competences *to be independent*, *to work jointly in a team*, and *to prioritize and make clinical decisions in a fast pace*.

#### To be independent

Being independent was described as being skilful, curious, engaged, positive, determined but at the same time careful, and reflective, while knowing one’s limitations. Independence brought a feeling of freedom regarding how to plan patient care and an ability to act swiftly to meet patient needs. With this feeling of freedom came great responsibility to amass sufficient knowledge and to question situations with potential to harm the patient. The nurses described playing a very central role in the ward; they felt like a “spider in a web”, pulling threads to provide the best care for each patient and that the PACU would strand without them. This was described by one of the nurses, as follows:

*as a nurse in the PACU, speaking generally now, I feel it’s a really important role – without us the whole unit would come to a stand-still* (interview no. 2)

Being independent involved thinking independently and possessing the knowledge needed to act on changes in the patients’ medical status based on written guidelines and general prescriptions for drugs. It also involved knowing what situations they could handle by themselves and when to call for assistance. The nurses said that they had to be able to act independently if an emergency arose when no physician was present in the ward. When the nurses had identified a situation in which they needed assistance, they not only called for assistance but also presented a possible solution to the problem.

*So then I contact the anaesthetist, ‘What’s the plan? Shouldn’t we administer a block instead,’ a sort of suggestion* (interview no. 7)

Several obstacles preventing the nurses from working independently were described. One of them was that the nurses could access only a few generally prescribed drugs, which inhibited them from swiftly meeting patient needs, limiting their ability to act on demand and work independently. The lack of a physician in the PACU was also said to complicate their work, since the nurses had to contact a physician by phone to get drug prescriptions, which could make a simple task very time consuming. In addition to the time wasted when contacting a physician, the nurses also believed that an oral drug prescription was riskier than a written one. The nurses stated that oral prescriptions could lead to misinformation or lost information, compromising patient safety.

#### To work jointly in a team

The nurses described how, during their working day, they collaborated closely not only with PACU colleagues such as nurses, assistant nurses, and physicians, but also with personnel working in the operating room (i.e., registered nurse anaesthetists [RNAs] and operating room nurses).

*you have to have a plan for the patient, plus, well, in collaboration with the anaesthetist of course, particularly when you’re dealing with critically ill patients* (interview no. 7)

The nurses tried to create joint plans with the patients regarding what would happen during their PACU stay, in order to build nurse–patient collaboration and a feeling that they were a team working together towards a common goal.

Good relationships and well-functioning communication were described as prerequisites for working jointly in a team, while hierarchical structures accomplished the opposite, inhibiting teamwork. Having different competences in the team, for example, a mix of intensive care nurses, RNAs, and nurses with post-graduate education in paediatrics or surgery, was believed to deepen team knowledge of different surgical procedures. If a nurse possessed specific knowledge and shared it with the other team members, this was said to enhance the knowledge of all co-workers and increase the overall competence of the team when working together.

The nurses stated that they needed to have “split vision” and be vigilant in order to give their colleagues help when needed. Supporting novice colleagues, both nurses and physicians, was believed to be highly demanding for the more experienced nurses working in the PACU. Superintending novice nurses to ensure that the patients received proper care and trying to contact physicians absent from the PACU created additional work for the more experienced nurses, who sometimes felt that they were neglecting their “own” patients.

#### To prioritize and make clinical decisions in a fast pace

When working in the PACU, the nurses said that they needed to keep up the pace throughout their working hours since there was a constant inflow of patients arriving from surgery. To make room for and take care of the patients who had undergone surgery, there had to be the same constant outflow of patients from the PACU to the surgical wards, or in some cases to the patients’ homes.

*when you have, when you’re at full capacity in the PACU, if we say that you…maybe four patients in, in rotation all the time, and then you have time to call someone [to the ward] and then the next one [patient] comes and you have time to send one [to the ward], it’s this that ends up being, this situation with juggling many things at once* (interview no. 2)

The nurses had to coordinate staff and patients so that the patient flow was optimized. This meant that the nurses had to work in a fast pace and stabilize patients as quickly as possible, without compromising the safety level of the care given.

*‘How can I get this patient ready to be sent home?’ Because I’m planning for the discharge right from the moment of arrival: ‘How are the patient and I going to manage this? How can we get this done today?’* (interview no. 4)

The constant patient flow required that the nurses had to simultaneously care for patients in different stages of recovery who had undergone different types of surgery and had different comorbidities. Since the pace was fast, the nurses found that they only had time for short, intensive meetings with each patient and that they had to gain an overview of patient medical status “in a blink of an eye”. The nurses believed that it was difficult to gain an overview of each patient in this limited time, and that the fast pace made it difficult to completely monitor all patients in their care. To simultaneously care for several patients, all with individual needs, the nurses developed different strategies, such as writing notes regarding when different drugs were to be administered, what and when different nursing activities were to be conducted, etc.

In one of the PACUs, the nurses rotated between the PACU and the preoperative unit, closely collaborating with each other. This made it possible for the nurses in the preoperative unit to intercept anything that might compromise optimal patient flow in the PACU. Sharing this information with the PACU nurses and preparing them for any eventualities allowed the PACU nurses to create strategies to meet this demand, thereby keeping up the patient flow.

The high patient flow sometimes resulted in a work situation that the nurses considered stressful and threatening to patient safety. The work situation was sometimes said to be very stressful and rushed, and the nurses described not having time to comply with written guidelines or evidence-based medicine and deviating from established routines.

### Creating safe care

The nurses working in the PACUs described having *to acknowledge and reassure the patient*, *to possess specific knowledge*, and *to anticipate the future and be proactive* as competences to create safe care for the patients in their care.

#### To acknowledge and reassure the patient

Caring for patients in the PACU required that the nurses acknowledge and reassure the patients. The patient meetings were described as short and intimate, with the patients believed to be in a vulnerable situation. The nurses tried to put themselves in the patients’ situation and treated the patients as they would like to be treated themselves.

*And then I think you also have to ask yourself ‘How would I like to be treated? If I was the one lying there in the bed, how would I like to be received and treated?’ I think that will tell you a lot about how you need to behave* (interview no. 5)

The experience of undergoing surgery is unique to each patient, and the nurses believed it could be intimidating, especially for children. This was the case regardless of the extent of the surgical procedure. The nurses therefore believed that they had an important task in showing the patients respect by maintaining the patients’ integrity, focusing on each patient, and adapting the care to each patient’s individual physical and psychological needs. Some of the patients’ physical and psychological needs were met when the nurses provided pain relief, ear plugs, a radio, music, and help relaxing. The nurses acknowledged the patients by means of non-verbal communication when staying near them, looking them in the eye, stroking them on the arm, or holding their hand. This was done to create a feeling of security and reassure the patient that the nurse was available, knew what to do, and would provide care.

Another aspect of creating a feeling of security was to provide patients with information in an understandable and neutral way. Since the patients often were unconscious and had not fully recovered from anaesthesia when arriving at the PACU, the nurses repeatedly provided them with the same information. The amount of information given was adapted to the depth of information the patient wanted to receive. The nurses believed that the many different languages spoken by patients could pose a real challenge, both when trying to provide the patients with information and when obtaining information from them, causing communication difficulties and thereby compromising patient safety.

It was challenging to meet the needs of patients who had been misinformed before surgery and, for example, had the wrong expectations regarding the surgery outcome.

Due to the patients’ unconsciousness, the nurses stated that they sometimes had to speak up on behalf of the patients and take the patients’ side, advocating for them.

#### To possess specific knowledge

Working in the PACU required that the nurses possess specific knowledge. Caring for patients in the PACU meant caring for patients who had undergone various surgeries, either in outpatient or inpatient care, including patients ranging from no comorbidities to multi-organ failure. The work involved caring for patients of all ages from infants to the elderly.

The PACU nurse’s role is wide ranging and therefore considered complex. This means that nurses working in PACUs must possess a great deal of specific knowledge about, for example, pain management, pharmacology, treatment of postoperative nausea and vomiting, medical techniques, different types of surgery together with early signs of complications, ECG interpretation, how drugs administered under anaesthesia could interact with drugs administered in the PACU, monitoring vital signs, physiology, anatomy, airway management, circulation, and fluid balance.

*And my work there entails to have knowledge about the surgery, the anaesthesia and postanaesthesia nursing, being able to identify, ‘diagnose’, and treat…complications related to the anaesthesia or the operation…I would say. And even be able to determine whether the patient is sufficiently okay…in terms of respiration, circulation, pain, etc.* (interview no. 11)

In addition, the nurses also must adapt the care given to each patient’s individual needs, take care of the patient’s next of kin, comfort parents of children who underwent surgery, meet various multicultural needs, among many other tasks.

Possessing specific knowledge involved knowing subtle things that are sometimes hard to put into words; this “tacit knowledge” can only be learned through prolonged work as a PACU nurse.

*I come back to this topic of the clinical gaze, being able to fairly quickly scan the room and observe the patients. And of course it’s just this, I mean the experience of…you get a feeling for it, so you know what you need to look for* (interview no. 16)

Some nurses said that postgraduate education in anaesthetic, intensive, or acute care was a prerequisite for PACU work, whereas others stated that it was sufficient to have a nursing degree. Some also claimed that nurses without a postgraduate diploma required prolonged training and skills development in the ward before they could independently care for their “own” patients, maintaining patient safety and keeping the patients from harm. Working with colleagues who lacked postgraduate education or previous PACU experience was described as threatening patient safety. This was because nurses without postgraduate education lacked the proper training and would not know what to expect. They might think that everything is fine and that the patient is being properly cared for, while the actual situation is exactly opposite, one nurse stated.

*‘What you don’t know, you don’t know. And what you don’t know, you also don’t know what to ask about’ – and that’s one of the great differences between having basic training and actually having a certain theoretical competence, but still having considerable knowledge about these sick patients, like ‘What is it I should be looking for, what is it I…’, I mean, ‘…when should I pull the hand brake, when should I sound the alarm?’* (interview no. 16)

The nurses believed that patient wellbeing might be at risk when critically ill patients who should be treated in the intensive care unit were transferred to the PACU due to lack of space. This was because PACU nurses might not possess either the knowledge or the experience to care for such patients.

#### To anticipate the future and be proactive

The nurses started planning a patient’s care and began working proactively as soon as the patient arrived in the PACU, during the handover from the anaesthetic personnel. Sometimes the nurses started planning even before the patient arrived, reading the patient’s medical records to find out what surgery had been conducted and identifying co-morbidities or other factors that might affect the patient’s recovery. The nurses stated that it was important to be “one step ahead” and prepared for any eventuality. They said that they needed to be vigilant, anticipating complications and being prepared for anything might happen; as one nurse said: “*You should know that something is about to happen even before it has happened*” (interview no. 14). After a patient’s arrival in the PACU, the nurses combined the information collected beforehand with that given by the anaesthetic personnel to create a complete overview of the patients’ medical status and wellbeing.

When caring for patients, the nurses kept themselves updated by continuously evaluating the patients’ physical status to be prepared for what was coming next. If the nurses identified any changes in patient status, they re-evaluated and, if needed, changed and adapted patient care based on each patient’s individual needs.

*So that, I mean, a lot of that postoperative stuff, I think the thing is you need to be one step ahead all the time. And to be able to do that effectively, of course you need to know ‘What is it I’m looking at, what is actually happening?’ because this area, it’s a matter of special medications special ingredien… – you need to understand the surgery, you need to understand the individual who has been subjected to it – ‘What does that individual bring to the picture?’ You need to have an advanced understanding of the relevant pharmacology, in a whole different way, there are many parameters, but also that everything affects everything else.* (interview no. 15)

The nurses stated that there were too many novice nurses relative to the number of patients passing through the PACU. This was said to interfere with the nurses being “one step ahead”, since they could not care for patients properly and could not perform all required tasks, thereby affecting patient safety.

*‘So it’s also this idea of always being one step ahead, like we do in the ICU, we’re a step ahead and that usually makes for fairly calm nursing care, it doesn’t get extremely complicated, you don’t get this – many people say ‘But this isn’t that urgent’ and ‘No, it’s because I’m always one step ahead, there are never any problems and that’s the best way to do things’.* (interview no. 9)

### Seeing the bigger picture

An expert PACU nurse was described as *seeing the bigger picture*. To do so, the nurse had to possess all of the required knowledge and unique skills required to work as a nurse in a Swedish PACU, as described in the themes *being adaptable in an ever-changing environment* and *creating safe care* combined with tacit knowledge. This meant that an expert nurse must be able to act independently, work jointly in a team, keep up the pace, acknowledge and reassure the patient, possess specific knowledge, and be able to anticipate the future and be proactive. In doing so, the expert nurses were described as able to adopt a “helicopter perspective”, taking all the aforementioned skills into account before deciding what to do in a given situation.

The expert nurses were described as very important for patient care as well as for their nurse colleagues and all other PACU staff. They knew what skills they did or did not possess, and would never perform a task they were uncertain of without advice from someone knowledgeable. They often passed their solid knowledge to their colleagues. The expert nurses supported their colleagues and were the ones others could always turn to when in need. Even in the most chaotic situations the expert nurses stood strong, calming the situations by being supportive and helping prioritize the most urgent task(s).

*you just have to look up and there’s that person saying ‘I’ll call’, they know exactly, because they see the patient, they see you, they can read both their colleague and the colleague’s patient, they have a clinical gaze that is somehow distinctive. They are also the ones who know the routines, they can navigate the nursing apparatus, the systems - they sort of know, they know the on-calls, they know…These are the people who…they also understand their own limitations, and there’s no ego in it..* (interview no. 15)

They were described as humble, empathic, warm, and present, and said to possess an inner calm that they conveyed to patients and colleagues by acknowledging them. The expert nurses were believed to keep up to date on the latest research, being eager to gain knowledge and be able to develop the care given (Fig. 1).

## Discussion

To our knowledge, this is the first study describing the competence required of nurses working in PACUs as well as the characteristics of expert nurses from the nurses’ perspective. The identified themes *being adaptable in an ever-changing environment*, *creating safe care*, and *seeing the bigger picture* show that nurses are central to PACU work, must be independent, and must have sufficient knowledge to provide safe and person-centred care to patients in a vulnerable situation. Nurses must acknowledge and reassure the patients, plan, grasp the situation, collaborate with the team, and work proactively to handle the shifting demands in the PACU due to workflow and patient acuteness.

*To possess specific knowledge* was important in providing safe care in PACUs. This could be a challenge if nurses working in PACUs have limited experience or no postgraduate diploma. To ensure safety, participants said that nurses without a postgraduate diploma needed more support and rigorous training. Nurses working in PACUs in Sweden must master various advanced technical skills [13]. Newly graduated nurses have said that they need continued training and that they lack the competence to master all the required technical skills [18]; they also report lower self-assessed competence [19]. Handling medications independently is an important technical skill in PACUs, and nurses without a postgraduate diploma report limited knowledge of medications and their side-effects and interactions [19]. A lack of sufficient technical skills could explain why it might be challenging to practice in the PACU as an inexperienced nurse, as described in our findings.

It is also possible that the challenges described by the nurses may be due to insufficient nontechnical skills and, more specifically, inadequate clinical reasoning (CR). Situational awareness, task management, and decision making can be seen as parts of CR. They are also three of the four categories of nontechnical skills identified in RNAs [8] and are also found in our study. Clinical reasoning, or other terms used synonymously are clinical judgment or decision making [20], is the process whereby nurses gather significant information, cues and relate this to theoretical knowledge to understand and plan how to handle a situation. Based on the outcome of the situation, the nurses also reflect on and learn from the process. Nurses with insufficient CR skills may fail to diagnose and react to certain situations, which has a significant impact on patient outcomes. CR is partly an unconscious process and includes intuitive and automatic cognitive processes [21] as well as tacit domain-specific knowledge [20] that is characteristic of expert nurses [5, 22, 23]. CR requires sufficient knowledge, education, skills, and experience [20, 21]. Finding the right cues depends on deep knowledge of physiology, pathophysiology, and pharmacology [21]. Nurses at lower competence levels have limited CR skills as they identify fewer cues and identify problems later [20, 21, 24]. In studying nurses caring for postoperative patients in the intensive care unit, Hoffman et al. showed that expert nurses worked more proactively, gathered more cues from more sources, and clustered cues in a more complex way than did novice nurses. Novice nurses were more reactive and acted later than did expert nurses [24]. Working proactively was also identified in our findings as a specific competence needed to provide safe care in PACUs. It has also been highlighted by Sundqvist et al. [25], Lyk-Jensen et al. [8], and Nilsson and Jaensson [26] in anaesthetic nursing, and by Björn and Lindberg Boström [27] in operating room nurses. This suggests that working proactively is a core competence of nursing in the perioperative context, which may be due to the fast pace, rapid change, and risk of adverse events possible in the anaesthetic and surgical context. We therefore believe that such deep knowledge and CR ability call for nurses with a higher level of education and experience.

Being an expert PACU nurse was interpreted as *seeing the bigger picture*. Proficiency in the unique PACU nursing skills, having specific and tacit knowledge, and seeing and acknowledging the patient gave nurses the ability to see the whole picture and handle situations in a timely way. Expert nurses were important for sharing knowledge, supporting the team around the patient, and developing care, as described by Morrison and Symes [22]. Characteristics of expert nursing practice were identified as *knowing the patient*, *intuitive knowledge*, *reflective practice*, *risk taking*, and *skilled know-how.* They also highlighted the importance of environmental factors, including positive relations with patients, physicians, and colleagues, and having expert nurses as role models [22], as also highlighted by Benner [5]. Benner described expert nurses as having appropriate education and experience [5] and as combining skills and knowledge in order to give complex and holistic care [28, 29]. A holistic perspective and seeing the whole patient is also needed by expert nurses handling postoperative pain to gain an overview of patients and manage their pain [24, 30]. The expert nurse can grasp and distinguish clinical issues and handle situations in a timely way because they act based on intuition and unconscious processes [5, 23, 29]. Such intuitive or unconscious processes were not explicitly described by the nurses in our study. However, for nurses to see the bigger picture and be supportive, give advice, share knowledge, and be confident and calm in all kinds of situations in the PACU, much of the competence would arise from unconscious processes and actions.

Furthermore, Benner highlighted the importance of defining what an expert nurse is, as it is context specific. This would make it clear to nurses at lower competence levels what skills are needed to reach higher levels of competence, as doing so is challenging if these skills are undefined [5]. This is supported by the present results, as more experienced nurses stated that their less experienced colleagues or colleagues without postgraduate education lacked theoretical knowledge and did not know what to expect. This is illustrated by one quotation in our findings: “*What you don’t know, you don’t know. And when you don’t know, you don’t know what to ask about either’*”.

As described above, the findings clearly identify a need for specific knowledge to provide safe PACU care. Nurses described a need for diverse knowledge and experience to support each other. Furthermore, the need for specialist nurse education was noted, and specialist nurse education in intensive care, surgical care, or anaesthetic care was suggested. The specific knowledge connected to these specializations was sometimes considered insufficient for the PACU context, and there was a desire for a nursing specialization in postoperative care. A desire for postoperative care education in Sweden has also been expressed by PACU nurse managers [13]. Several studies from Europe and USA have demonstrated that higher education in nurses positively affects outcomes in patients undergoing surgery [31–36], and that experience per se may not replace education in improving patient outcomes [35, 36]. Although this was not studied specifically in the PACU context, it is reasonable to think that this finding is transferable to nurses working in PACUs, as it was expressed by the participants in our study.

Knowledge from this study can be used to define the scope of practice [4] of nurses working in PACUs in Sweden. It can also form guidelines for skill acquisition by nursing students and nurses at lower competence levels. As there is no education specifically for nurses working in PACUs in Sweden, the present results together with those of two other studies by the same authors [12, 13] could form the basis for developing education for PACU nurses in Sweden.

### Methodological considerations

There are both strengths and limitations of this study that should be highlighted. Interviewing both male and female nurses from two PACUs in Sweden with a broad range of work experience might have contributed to rich descriptions, enhancing the credibility of the results [37]. An interactive process between all authors involved discussing the analysis until consensus was reached [37]. The credibility of the results was further enhanced by citing examples of how the analysis emerged from the data extracts via codes, sub-themes, and finally themes, and by citing representative quotations to allow the reader to judge whether the analysis was reasonable in relation to the raw data [38]. The interviews were transcribed by a professional transcriptionist; the transcripts were then read through by one researcher who simultaneously listened to the recorded interviews, increasing the credibility of the results [17]. In the research group there were mixed pre-understandings: two researchers have experience working in PACUs as RNAs, one researcher has experience working in a PACU, and one has experience working as an RNA. Two researchers are senior researchers, which, together with their multidisciplinary roles, may be a strength of the study, increasing the credibility of the results [38].

To be consistent when collecting data and thereby increasing the dependability of the results, an interview guide was used, and we described in detail how the data collection and analysis process was conducted. It is important that the results reflect the informants’ experiences and not the researchers’ perceptions, and to this end, the use of recordings increased the confirmability of the results [38]; however, determining the results’ transferability rests with the reader [39]. The patterns identified from the interviews depend on the context, which in turn influences the transferability of the results [38]. Demographic data were therefore provided so that the reader can draw conclusions on similarities between the study context and that to which the results are to be transferred.

A limitation was that the interviews might have been affected by having been conducted by a researcher with the same profession as the interviewees (i.e., a PACU nurse). The nurses might have felt safe sharing their views with an interviewer from the same profession using the same vocabulary; on the other hand, some nurses might have decided not to share their experiences because of a sense of shame or failure, i.e., socially desirable responding [40]. However, the nurses apparently willingly shared their personal views regarding their professional role and situations in which they had felt challenged, possibly decreasing the risk of the results being influenced by socially desirable responding.

The data was analysed in relation to the two aims and presented as separate themes in the findings. It can be argued that they should be interpreted and presented together since being an expert nurse also includes the competences described in the first two themes. However, in relation to the importance of defining an expert nurse the authors made the choice to report these findings in a separate theme.

This is an area with limited research. Our findings are based on nurses’ perceptions, further studies should include diverse methodologies and research from PACUs in other countries.

## Conclusion

The PACU is an ever-changing environment with a wide diversity of patients with individual needs. Nurse competence in the PACU requires specific knowledge, acknowledging the patient, and working proactively at a fast pace with the patients and team to provide safe, high-quality care. An expert PACU nurse possesses all the required specific competences and can see the bigger picture, helping share knowledge and develop PACU care. The expert competence of seeing the bigger picture can be used in supervising novices and creating a knowledge base for postgraduate education in order to promote safe, high-quality care in PACUs.

## Data Availability

The datasets generated and analysed during the current study are not publicly available due to the participants confidentiality and the researchers have no ethical permission to share them. The corresponding author can be contacted if someone wants to request the data from this study.

## Abbreviations

CR: Clinical reasoning
PACU: Post-anaesthesia care unit
RN: Registered nurse
RNA: Registered nurse anaesthetist
